# In-Depth Bioinformatic Study of the CLDN16 Gene and Protein: Prediction of Subcellular Localization to Mitochondria

**DOI:** 10.3390/medicina55080409

**Published:** 2019-07-26

**Authors:** Erasmia Rouka, Vassilios Liakopoulos, Konstantinos I. Gourgoulianis, Chrissi Hatzoglou, Sotirios G. Zarogiannis

**Affiliations:** 1 Department of Transfusion Medicine, University Hospital of Larissa, Biopolis, 41334 Larissa, Greece; 2 Peritoneal Dialysis Unit, 1st Department of Medicine, Ahepa Hospital, Aristotle University of Thessaloniki, 54636 Thessaloniki, Greece; 3 Department of Respiratory Medicine, Faculty of Medicine, University of Thessaly, Biopolis, 41334 Larissa, Greece; 4 Department of Physiology, Faculty of Medicine, University of Thessaly, Biopolis, 41500 Larissa, Greece

**Keywords:** bioinformatics, CLDN16, FHHNC, interactome, mitochondria, subcellular localization

## Abstract

*Background and Objectives:* The defects in the *CLDN16* gene are a cause of primary hypomagnesemia (FHHNC), which is characterized by massive renal magnesium wasting, resulting in nephrocalcinosis and renal failure. The mutations occur throughout the gene’s coding region and can impact on intracellular trafficking of the protein or its paracellular pore forming function. To gain more understanding about the mechanisms by which *CLDN16* mutations can induce FHHNC, we performed an in-depth computational analysis of the CLDN16 gene and protein, focusing specifically on the prediction of the latter’s subcellular localization. *Materials and Methods:* The complete nucleotide or amino acid sequence of CLDN16 in FASTA format was entered and processed in 14 databases. *Results:* One CpG island was identified. Twenty five promoters/enhancers were predicted. The *CLDN16* interactome was found to consist of 20 genes, mainly involved in kidney diseases. No signal peptide cleavage site was identified. A probability of export to mitochondria equal to 0.9740 and a cleavable mitochondrial localization signal in the N terminal of the CLDN16 protein were predicted. The secondary structure prediction was visualized. Νo phosphorylation sites were identified within the CLDN16 protein region by applying DISPHOS to the functional class of transport. The KnotProt database did not predict any knot or slipknot in the protein structure of CLDN16. Seven putative miRNA binding sites within the 3’-UTR region of CLDN16 were identified. *Conclusions:* This is the first study to identify mitochondria as a probable cytoplasmic compartment for CLDN16 localization, thus providing new insights into the protein’s intracellular transport. The results relative to the *CLDN16* interactome underline its role in renal pathophysiology and highlight the functional dependence of CLDNs-10, 14, 16, 19. The predictions pertaining to the miRNAs, promoters/enhancers and CpG islands of the *CLDN16* gene indicate a strict regulation of its expression both transcriptionally and post-transcriptionally.

## 1. Introduction

The *CLDN16* gene is clustered on chromosome 3q28 and encodes the claudin16 protein which is found primarily in the kidneys, specifically in the thick ascending limb (TAL) of the loop of Henle where it regulates the paracellular resorption of magnesium ions. The defects in the *CLDN16* gene are a cause of primary hypomagnesemia (FHHNC) (OMIM # 248250; HOMG3), which is characterized by massive renal magnesium wasting with hypomagnesemia and hypercalciuria resulting in nephrocalcinosis and renal failure (Entrez gene summary: https://www.ncbi.nlm.nih.gov/gene/10686). The mutations in the gene encoding the claudin19 protein can also result in the aforementioned renal disorder although, in this case, the disease phenotype is also complicated by ocular involvement (OMIM # 248190; HOMG5). The renal prognosis for both types is poor, with progressive chronic kidney disease requiring renal replacement therapy usually presenting in the second or third decade of life [1].

The mutations in *CLDN16* and *CLDN19* occur throughout the coding region and can affect proper folding, intracellular trafficking of the protein or its paracellular pore forming function [2]. Regarding, in particular, the impact of *CLDN16* mutations on protein function, a genotype/phenotype correlation has been proposed reflecting the severity of FHHNC [3]. The possible contribution of epigenetic factors or genetic modifiers on the phenotypic variability observed in some patients with FHHNC has also been proposed, although no studies have addressed this hypothesis [4].

More than 50 pathogenic mutations have been reported so far, including mainly missense/nonsence mutations, splice-site mutations and small deletions of the *CLDN16* gene, yet the pathophysiology of the disease remains obscure [4]. It has been shown that disease-associated mutations affect either the intracellular traffic of CLDN16 or its capacity to facilitate paracellular Mg^2+^ transport [5]. The mutant CLDN16 molecules of the first category accumulate in different intracellular compartments of the exocytic and/or endocytic pathways while those included in the second category are correctly delivered to the tight junction (TJ) but are defective in Mg^2+^ permeability [5]. It has been reported that most of mutations in the *CLDN16* gene of FHHNC patients and dephosphorylation of CLDN16 induce abnormal cytoplasmic localization [6].

Notably, the CLDN16 mutants which are distributed in the TJ have full or partial function in contrast with the ones that are mislocalized to the cytoplasmic compartments (endoplasmic reticulum, Golgi apparatus, lysosomes) which are not functional [6]. Although the mislocalization of CLDN16 leads to loss of its function, the mechanism of its transport has not yet been clarified [6].

Taking into account the above observations, this study aimed to perform an in-depth computational analysis of the CLDN16 gene and protein, focusing specifically on the prediction of the latter’s subcellular location in order to gain a better understanding about the mechanisms by which *CLDN16* mutations can induce renal diseases.

## 2. Materials and Methods

The nucleotide sequence of the *CLDN16* gene was downloaded in FASTA format from the Ensembl database. The EMBOSS_CpGplot tool [7] was employed to identify putative CpG islands using the criteria by Takai and Jones [8]: Observed/expected ratio >0.65; percent C + percent G >55.00; length >500. 

The prediction of promoters and enhancers was performed by the FPROM database [9]. The GeneMania database was used for the prediction of the *CLDN16* gene interactors [10]. The description of the retrieved genes was provided by the GeneCards database [11]. The functional enrichment analysis of gene ontology (GO) annotations relative to disease of the *CLDN16* interactome was performed using ToppFun, an application of the ToppGene Suite [12].

Similarly, the amino acid sequence of the CLDN16 protein was downloaded in FASTA format from the Uniprot database (UniProtKB ID: Q9Y5I7). The ProtParam tool was used for the prediction of the protein’s physicochemical properties [13]. The SignalP v.4.1 Server carried out the prediction of the presence and location of signal peptide cleavage sites [14]. The combined transmembrane topology and signal peptide prediction was performed by the Phobius database [15]. The protein subcellular location was predicted by WoLFPSORT [16]. MitoProt II and Mitofates were used for the identification of putative mitochondrial targeting sequences and cleavage sites [17,18]. The prediction of the secondary structure was performed by the PSIPRED v.3.3 database as the latter also provides the possibility of identifying disordered protein regions through the disorder predictor tool DISOPRED2 [19]. The prediction of protein knots was conducted using the KnotProt v.2.0 database [20]. The DISPHOS v.1.3 database was used to predict serine, threonine and tyrosine phosphorylation sites within the CLDN16 protein selecting transport as the latter’s functional category [21].

The identification of predicted and validated *CLDN16*-miRNA targets was performed by the miRWalk v.2.0 database using the default parameters (3’UTR, start position of miRNA seed=position 1, minimum seed length=7) [22].

**Figure 1:**
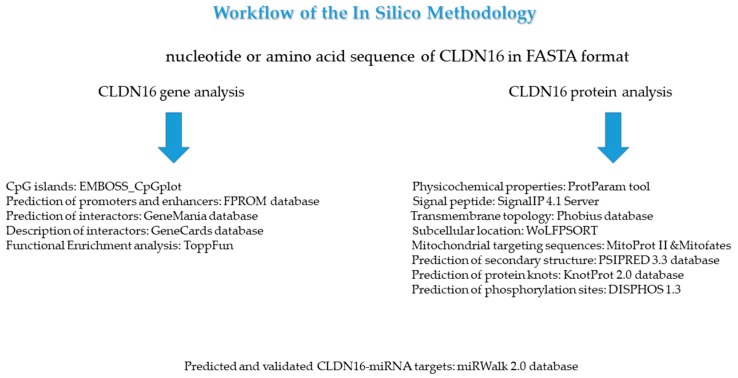
The display of the in-silico methodology steps that were applied in this study.

The selection of the aforementioned bioinformatic tools was made mainly on the basis of two factors: Performance and quality of documentation. All analyses were performed on September 2018. The specific versions of the databases have been indicated in the cases where more than one version is available. The workflow of the in-silico methodology used in this study is visualized in Figure 1.

## 3. Results

### 3.1. CLDN16 Gene

**Figure 2:**
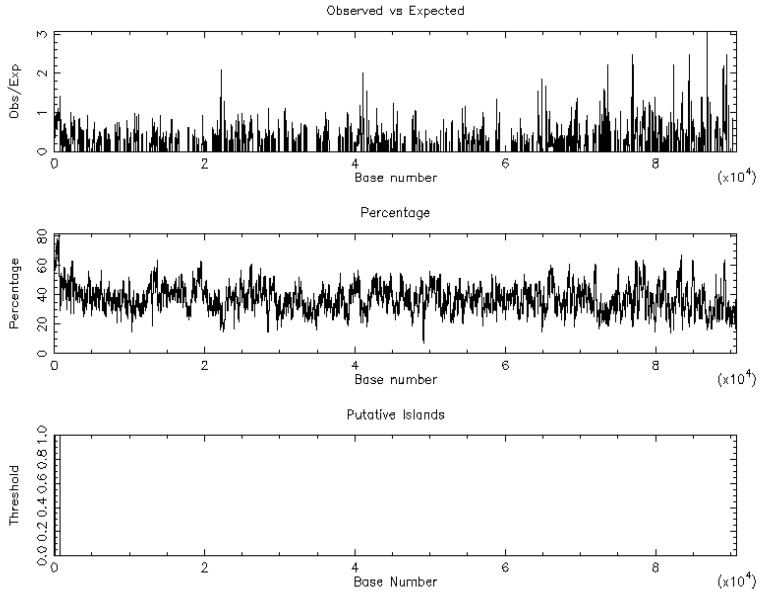
The CpG island graph of the *CLDN16* gene as retrieved by the EMBOSS_CpGplot tool using the criteria: Observed/Expected ratio >0.65; percent C + percent G >55.00; length >500. One island of unusual CG composition was identified (from 87–712, length 626).

One CpG island was identified in the nucleotide sequence of the *CLDN16* gene (Figure 2). Twenty five promoters/enhancers were predicted in the FPROM analysis (Table 1). The GeneMania database identified 20 genes as possible interactors of the *CLDN16* gene (Table 2). According to ToppFun, the *CLDN16* interactome is mainly involved in kidney disorders (Table 3).

### 3.2. CLDN16 Protein

**Figure 3:**
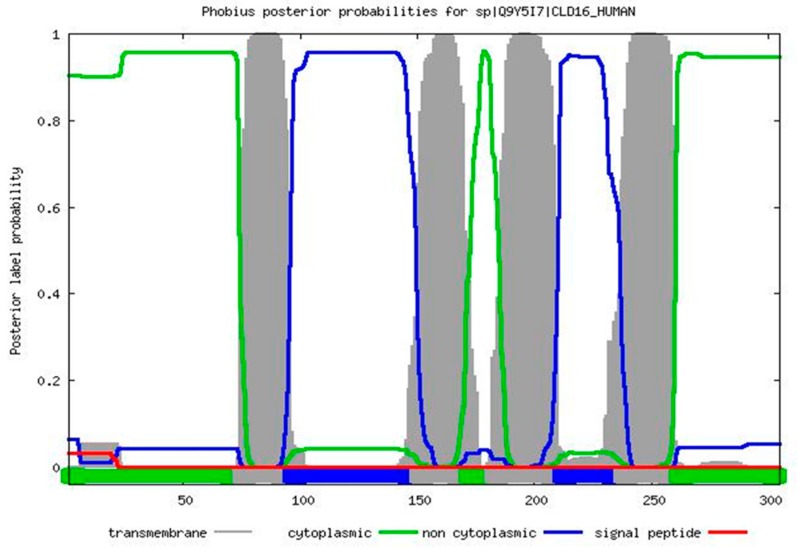
The results of the combined transmembrane topology and signal peptide prediction analysis as retrieved by the Phobius database.

**Figure 4:**
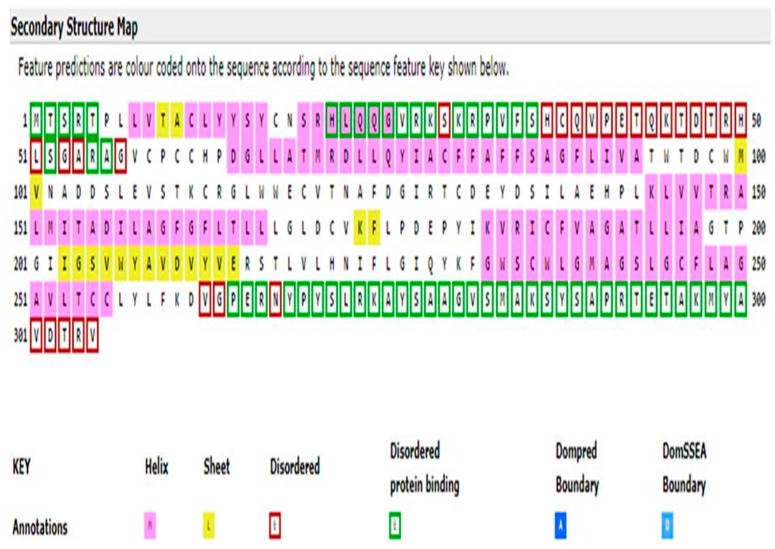
The secondary structure map of the CLDN16 protein as predicted by the PSIPRED v.3.3 software. Τhe intrinsic disorder predictor DISOPRED2 was also used to identify disordered regions.

**Figure 5:**
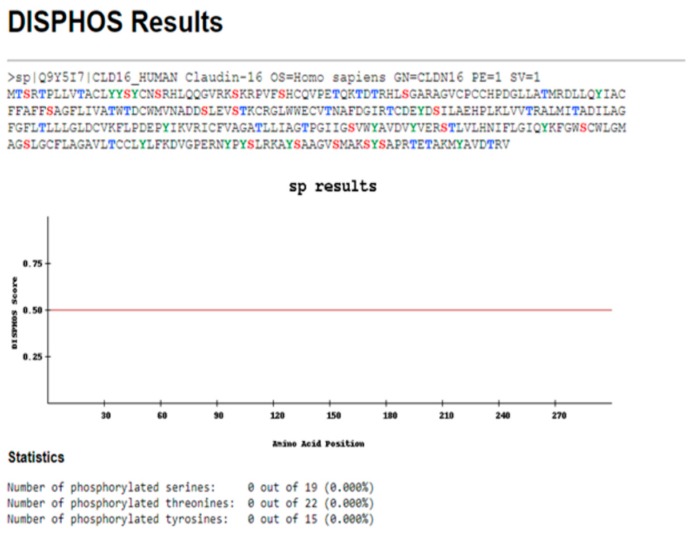
The results obtained by the DISPHOS 1.3 database regarding the prediction of phosphorylation sites within the CLDN16 protein selecting transport as the latter’s functional class.

The ProtParam Tool predicted that the CLDN16 protein has a theoretical isoelectric point and an instability index equal to 8.26 and 35.14 respectively. The latter value classifies the protein as stable. No signal peptide cleavage site was identified by the SignalP v.4.1 Server and the Phobius database. The results of the combined transmembrane topology and signal peptide prediction analysis as retrieved by the latter database are presented in Figure 3. The WoLFPSORT database identified 32 nearest neighbors (plasma membrane: 19, extracellular: 8, mitochondrial: 2, nuclear: 1, peroxisomal: 1, golgi: 1). MitoProt II predicted a probability of export to mitochondria equal to 0.9740 while Mitofates identified a cleavable localization signal in the N terminal of the CLDN16 protein (22 MPP cleavage site). The secondary structure prediction is shown in Figure 4. The KnotProt v.2.0 database did not predict any knot or slipknot in the protein structure of CLDN16. Νo phosphorylation sites were identified within the CLDN16 protein region by applying DISPHOS 1.3 to the functional class of transport (Figure 5).

### 3.3. miRNA Analysis

The miRWalk v.2.0 database predicted that *CLDN16* was most probably a target of the miRNAs presented in Table 4.

## 4. Discussion

In this study we aimed to perform an in-depth computational study of the CLDN16 gene and protein so as to gain a better understanding about the latter’s involvement in the pathophysiology of renal disorders. Due to the fact that mislocalized CLDN16 mutants lose their function [6], this study focused specifically in the prediction of the protein’s subcellular location. Our results, which have been reproduced in two independent databases, are the first to identify mitochondria as a probable cytoplasmic compartment for CLDN16 localization, thus providing new insights into the protein’s intracellular transport. Additionally, no signal peptide cleavage site was identified. The integral membrane proteins carry a signal peptide and/or a transmembrane domain that mediates their insertion into the endoplasmic reticulum from where they exit to reach the Golgi apparatus and the plasma membrane [23]. Two questions arise from the above findings: 1. What is the functional role of the CLDN16 protein in mitochondria, assuming the latter actually locates at this site? 2. Which are the processes that control the protein’s translocations?

The regulation of protein trafficking relies on information that is encoded within the protein sequence and occurs by two major mechanisms, namely co-translational and post-translational translocation [24]. CLDN16 is located in the TJs but the mechanism regulating its localization is unclear [25]. It has been reported that in renal tubular epithelial cells the tight junctional localization of CLDN16 is regulated by Syntaxin 8 (STX8) [6]. In addition, the association between the two proteins requires the phosphorylation of CLDN16 [6]. The dephosphorylation of CLDN16 increases its intracellular distribution and decreases paracellular Mg^2+^ permeability [25]. The RING finger-and PDZ domain-containing protein PDZRN3 mediates the endocytosis of dephosphorylated CLDN16, thus representing an important component of the CLDN16-trafficking machinery in the kidney [25]. Notably, in this study, no phosphorylation sites were identified within the CLDN16 protein region by applying DISPHOS to the functional protein category of transport. This is important as DISPHOS uses disorder information to improve the distinction between phosphorylation and non-phosphorylation sites [21]. Based on both our findings and the existing literature, we could speculate that the dephosphorylated form of the CLDN16 protein may translocate to the mitochondria although cellular subfractionation studies need to be performed in order to prove this hypothesis. In addition, it would be of interest to see whether the protein as a whole or a fraction of it, as in the case of the retinoblastoma protein, reaches the mitochondria compartment [26].

The presence of mutations leading to defects in protein trafficking is an acknowledged pathogenetic mechanism observed in an increasing number of disorders, including approximately one third of monogenic diseases affecting the kidneys [27]. In the case of the FHHNC disease, various mutations in the *CLDN16* gene can lead either to the retention of the protein product in the endoplasmic reticulum and Golgi compartments or to its mislocalization to lysosome [27]. Notably, the *CLDN16* gene interaction network appears to be associated with Bartter syndrome type 4, which results from mutations in the *BSND* gene also affecting the trafficking and function of CIC-K channels [28,29]. The remaining results of the functional analysis are also intriguing as they link the *CLDN16* interactome with disorders of water, electrolytes and acid-base metabolism. The epithelial cells in the TAL, form a water-impermeable barrier, actively transport Na^+^ and CI^−^ via the transcellular route and provide a paracellular pathway for the selective reabsorption of Mg^2+^ and Ca^2+^ [30]. An interesting study relating to salt and acid-base metabolism in *CLDN16* knockdown mice, revealed that the loss of *CLDN16* results in increased urinary flow, reduced HCO3- excretion and lower urine pH [31].

The identification, in this work, of *CLDN19* and *CLDN14* as members of the *CLDN16* gene regulatory network denotes the functional interplay of these genes, which has been confirmed in previous studies [30,32]. It has been reported that the CLDN14 protein blocks the paracellular cation channel formed by the CLDN16-CLDN19 protein complex that is critical for Ca^2+^ reabsorption in the TAL [32]. Of interest, the gene expression of *CLDN14* is regulated on the post-transcriptional level by two microRNAs (miR-9 and miR-374) which directly target the 3′-UTR of the CLDN14 mRNA inducing its decay and translational repression [30,32]. The Ca^2+^ sensing receptor (CaSR) acts upstream of the microRNA-CLDN14 axis [32] providing thus a regulatory loop to maintain Ca^2+^ homeostasis in the kidney [30]. Another finding that should be commented upon, pertaining to the *CLDN16* gene interaction network, is the identification of the *CLDN10* gene as recently it was reported that deletion of the latter rescues *CLDN16*-deficient mice from hypomagnesemia and hypercalciuria [33]. It is worth noting that the four aforementioned *CLDN* genes have been included in a list of 16 genes (both differentially expressed and differentially methylated) which ranked in the top 15% of the nodes of an integrated gene regulatory network in kidney renal clear cell carcinoma [34]. We currently perform an in-silico transcriptomic analysis of the *CLDN16* interactome in kidney cancer to examine possible associations.

With respect to the miRNA analysis results, this study predicted seven putative miRNA bindind sites within the 3’-UTR region of CLDN16. The miRNAs partake in the regulation of almost every cellular process and are associated with many human pathologies including kidney diseases [35]. In the TAL of the loop of Henle, miRNAs not only regulate the Ca^2+^ metabolism as mentioned above, but also the salt and fluid handling [35]. This was evidenced in a study which demonstrated that the miR-192 suppresses the β-1 subunit of Na(+)/K(+)-ATPase, the enzyme that provides the driving force for tubular transport [36]. To our knowledge, no literature exists with respect to the miRNAs that have been identified in this study and FHHNC disease. Recently, a group from Spain standardized the protocol conditions for the identification of differentially expressed miRNAs in urinary exosome-like vesicles of FHHNC patients (and other renal diseases) characterized by polyuria [37].

The CpG islands are sites of transcription initiation [38] and have been characterized lately as “hotspots for global gene regulation” [39]. At the same time, promoters and enhancers are DNA regulatory regions accountable for ensuring proper spatiotemporal expression patterns of eukaryotic genes [40]. Most genes have multiple promoters and 72% of human promoters are associated with CpG islands [41]. The frequency of TATA box containing promoters among human protein-coding genes has been reported to be 10–20% with the result that the majority of protein-coding genes are regulated by TATA-less promoters [41]. The FPROM method which has been used in this study for the identification of potential transcription start positions has been shown to predict with high accuracy both types of promoters [9]. The identification in this study of seven miRNA binding sites along with the prediction of one CpG island and twenty five promoters/enhancers within the nucleotide sequence of the *CLDN16* gene provides the clue that the latter’s expression is possibly strictly regulated at the transcriptional and post-transcriptional level.

A limitation of our study was that the results are mainly based on predictions. Bioinformatics should be combined with experimentation to generate more accurate and reliable interpretations, however the in-silico analysis allows researchers to take an informed decision before proceeding in an expensive and time consuming experiment for further validation [42]. The bioinformatics tools that have been used in this study have been tested for their performance as evidenced by the publications mentioned therein.

## 5. Conclusions

This study performed a thorough bioinformatic analysis of the CLDN16 gene and protein. Our main finding is the prediction that mitochondria are a probable subcellular compartment for the localization of the CLDN16 protein. The conditions under which the CLDN16 protein (or a fraction of it) reaches this organelle along with its possible functional role there, must be further investigated at the experimental level. Our results with respect to the *CLDN16* interactome underline its role in renal pathophysiology and highlight the functional dependence of the *CLDN16*-*CLDN19*-*CLDN14*-*CLDN10* genes. The predictions pertaining to the miRNAs, promoters/enhancers and CpG islands of the *CLDN16* gene provide indications for a strict regulation of its expression both transcriptionally and post-transcriptionally. Our report inculcates the idea of studying both the potential translocation of the CLDN16 protein to mitochondria and the functional role of the *CLDN16* gene regulatory network in kidney disorders other than FHHNC.

## Figures and Tables

**Table 1:** The 25 potential transcription start positions predicted by the FPROM algorithm in the nucleotide sequence of *CLDN16*.

Promoter Position	49220 LDF ^1^: +9.953
Promoter Position	559 LDF: +6.606 TATA box at 527 +3.708 GATTTAAA; Enchancer at: 622 Score: +10.194
Promoter Position	58406 LDF: +5.233 TATA box at 58379 +4.957 TATATAGA
Promoter Position	40172 LDF: +3.884 TATA box at 40145 +8.509 TATAAAAC
Promoter Position	29232 LDF: +2.816 TATA box at 29200 +3.764 CATATAGA
Promoter Position	27968 LDF: +2.513 TATA box at 27936 +7.471 TATATAAG
Promoter Position	906 LDF: +1.626 TATA box at 865 +5.343 TATTAAAA; Enchancer at: 622 Score: +10.194
Promoter Position	41678 LDF: +1.619 TATA box at 41643 +4.703 TATATATA; Enchancer at: 41701 Score: +10.937
Promoter Position	3335 LDF: +1.403 TATA box at 3304 +5.535 TATATAAT
Promoter Position	48910 LDF: +1.227 TATA box at 48877 +6.554 AATATAAA
Promoter Position	44869 LDF: +0.663 TATA box at 44839 +4.130 TATAACAG
Promoter Position	65626 LDF: +0.437 TATA box at 65594 +5.926 TATATAAA
Promoter Position	69400 LDF: +0.423 TATA box at 69370 +7.797 TATAAAAG
Promoter Position	32529 LDF: +0.116 TATA box at 32500 +8.328 TATAAAAA
Promoter Position	51931 LDF: +0.008 TATA box at 51900 +3.876 TATTAAAA
Promoter Position	4793 LDF: −0.042 TATA box at 4762 +3.800 CATAAAAC
Promoter Position	81748 LDF: −0.140 TATA box at 81718 +4.492 CTTAAAAA
Promoter Position	27538 LDF: −0.398 TATA box at 27508 +5.750 TATAAATT
Promoter Position	50088 LDF: −0.458 TATA box at 50057 +5.632 CATATAAG
Promoter Position	67124 LDF: −0.489 TATA box at 67094 +3.638 TTTAAATC
Promoter Position	36521 LDF: −0.500 TATA box at 36492 +5.469 AATAAAAA
Promoter Position	52521 LDF: −0.572 TATA box at 52491 +4.197 CATAAAAG
Promoter Position	34329 LDF: −0.821 TATA box at 34301 +4.292 AATAAAAA
Promoter Position	68333 LDF: −0.879 TATA box at 68303 +4.256 TTTAAAGG
Promoter Position	85754 LDF: −0.905 TATA box at 85723 +5.966 TATTTAAA

^1^ LDF: Linear Discriminant Function: value of Fisher’s linear discriminant for the current promoter. A bigger value stands for more reliable promoter [9].

**Table 2:** The gene interaction network (interactome) of the *CLDN16* gene as retrieved by the GeneMania database.

Gene name	Gene Description
*CLDN19*	Claudin 19
*CLDN14*	Claudin 14
*CLDN10*	Claudin 10
*CLDN6*	Claudin 6
*TJP1*	Tight Junction Protein 1
*TJP3*	Tight Junction Protein 3
*NPHS1*	NPHS1, Nephrin
*PATJ*	PATJ, Crumbs Cell Polarity Complex Component
*MYO3B*	Myosin IIIB
*C14orf105*	Coiled-Coil Domain Containing 198
*CLCNKA*	Chloride Voltage-Gated Channel Ka
*CLCNKB*	Chloride Voltage-Gated Channel Kb
*CTXN3*	Cortexin 3
*FCAMR*	Fc Fragment Of IgA And IgM Receptor
*CDH16*	Cadherin 16
*LYG1*	Lysozyme G1
*FBXO40*	F-Box Protein 40
*KCNJ1*	Potassium Voltage-Gated Channel Subfamily J Member 1
*TMEM178B*	Transmembrane Protein 178B
*ADGRF3*	Adhesion G Protein-Coupled Receptor F3

The gene description has been provided by the GeneCards database.

**Table 3:** Functional enrichment analysis relative to disease of the *CLDN16* interactome. The results were obtained through ToppFun, an application of the ToppGene suite. FDR: False Discovery Rate; B and H: Benjamini-Hochberg; B and Y: BenjaminiYekutieli. Only the top 10 results are presented.

	ID	Name	Source	*p* Value	FDR B&H	FDR B&Y	Bonferroni	Genes from Input	Genes in Annotation
1	C1846352	Increased urinary chloride	DisGeNET Curated	7.888 × 10^−9^	1.037 × 10^−6^	6.376 × 10^−6^	2.074 × 10^−6^	3	5
2	C1846351	Increased urinary potassium	DisGeNET Curated	7.888 × 10^−9^	1.037 × 10^−6^	6.376 × 10^−6^	2.074 × 10^−6^	3	5
3	C0085680	Hypochloremia (disorder)	DisGeNET Curated	1.577 × 10^−8^	1.037 × 10^−6^	6.376 × 10^−6^	4.146 × 10^−6^	3	6
4	C0595901	Serum chloride level decreased (finding)	DisGeNET Curated	1.577 × 10^−8^	1.037 × 10^−6^	6.376 × 10^−6^	4.146 × 10^−6^	3	6
5	C1865279	Fetal polyuria	DisGeNET Curated	4.409 × 10^−8^	2.319 × 10^−6^	1.427 × 10^−5^	1.160 × 10^−5^	3	8
6	C1846347	Renal salt wasting	DisGeNET Curated	6.386 × 10^−7^	2.799 × 10^−5^	1.722 × 10^−4^	1.680 × 10^−4^	3	18
7	cv:C2751312	Bartter syndrome, type 4b	Clinical Variations	9.148 × 10^−7^	3.007 × 10^−5^	1.850 × 10^−4^	2.406 × 10^−4^	2	2
8	OMIN:613090	Bartter syndrome, type 4b	OMIM	9.148 × 10^−7^	3.007 × 10^−5^	1.850 × 10^−4^	2.406 × 10^−4^	2	2
9	C0740896	Hypokalemic hypochloremic metabolic alkalosis	DisGeNET Curated	2.740 × 10^−6^	6.226 × 10^−5^	3.830 × 10^−4^	7.207 × 10^−4^	2	3
10	OMIN:602522	Bartter syndrome, type 4a	OMIM	2.740 × 10^−6^	6.226 × 10^−5^	3.830 × 10^−4^	7.207 × 10^−4^	2	3

**Table 4:** The putative miRNA bindind site predictions within the 3’-UTR region of CLDN16. The results were obtained from the miRWalk v.2.0 database. Only results with the two smaller *p*-values are presented.

miRNA	Seed Length	*p*-Value
hsa-miR-6076	11	0.0005
hsa-miR-6878-3p	10	0.0020
hsa-miR-328-5p	10	0.0020
hsa-miR-559	10	0.0020
hsa-miR-1256	10	0.0020
hsa-miR-6859-5p	10	0.0020
hsa-miR-95-5p	10	0.0020

## References

[1] Viering D.H.H.M., de Baaij J.H.F., Walsh S.B., Kleta R., Bockenhauer D. (2017). Genetic causes of hypomagnesemia, a clinical overview. Pediatric Nephrol..

[2] Arteaga M.E., Hunziker W., Teo A.S.M., Hillmer A.M., Mutchinick O.M. (2015). Familiar hypomagnesemia with hypercalciuria and nephrocalcinosis: Variable phenotypic expression in three affected sisters from Mexican ancestry. Ren. Fail..

[3] Hanssen O., Castermans E., Bovy C., Weekers L., Erpicum P., Dubois B., Bours V., Krzesinski J.-M., Jouret F. (2014). Two novel mutations of the CLDN16 gene cause familiar hypomagnesaemia with hypercalciuria and nephrocalcinosis. Clin. Kidney J..

[4] Claverie-Martin F. (2015). Familiar hypomagnesaemia with hypercalciuria and nephrocalcinosis: Clinical and molecular characteristics. Clin. Kidney J..

[5] Kausalya P.J., Amasheh S., Günzel D., Wurps H., Müller D., Fromm M., Hunziker W. (2006). Disease-associated mutations affect intracellular traffic and paracellular Mg2+ transport function of Claudin-16. J. Clin. Investig..

[6] Ikari A., Tonegawa C., Sanada A., Kimura T., Sakai H., Hayashi H., Hasegawa H., Yamaguchi M., Yamazaki Y., Endo S. (2014). Tight junctional localization of claudin-16 is regulated by syntaxin 8 in renal tubular epithelial cells. J. Biol. Chem..

[7] Rice P., Longden I., Bleasby A. (2000). EMBOSS: The European Molecular Biology Open Software Suite. Trends Genet..

[8] Takai D., Jones P.A. (2002). Comprehensive analysis of CpG islands in human chromosomes 21 and 22. Proc. Natl. Acad. Sci. USA.

[9] Solovyev V.V., Shahmuradov I.A., Salamov A.A. (2010). Identification of promoter regions and regulatory sites. Methods Mol. Biol..

[10] Warde-Farley D., Donaldson S.L., Comes O., Zuberi K., Badrawi R., Chao P., Franz M., Grouios C., Kazi F., Lopes C.T. (2010). The GeneMANIA prediction server: Biological network integration for gene prioritization and predicting gene function. Nucleic Acids Res..

[11] Rappaport N., Fishilevich S., Nudel R., Twik M., Belinky F., Plaschkes I., Stein T.I., Cohen D., Oz-Levi D., Safran M. (2017). Rational confederation of genes and diseases: NGS interpretation via GeneCards, MalaCards and VarElect. Biomed. Eng. Online.

[12] Chen J., Bardes E.E., Aronow B.J., Jegga A.G. (2009). ToppGene Suite for gene list enrichment analysis and candidate gene prioritization. Nucleic Acids Res..

[13] Gasteiger E., Hoogland C., Gattiker A., Duvaud S., Wilkins M.R., Appel R.D., Bairoch A., Walker J.M. (2005). Protein Identification and Analysis Tools on the ExPASy Server.

[14] Nielsen H. (2017). Predicting Secretory Proteins with SignalP. Methods Mol. Biol..

[15] Käll L., Krogh A., Sonnhammer E.L. (2007). Advantages of combined transmembrane topology and signal peptide prediction—The Phobius web server. Nucleic Acids Res..

[16] Horton P., Park K.J., Obayashi T., Fujita N., Harada H., Adams-Collier C.J., Nakai K. (2007). WoLF PSORT: Protein localization predictor. Nucleic Acids Res..

[17] Claros M.G., Vincens P. (1996). Computational method to predict mitochondrially imported proteins and their targeting sequences. Eur. J. Biochem..

[18] Fukasawa Y., Tsuji J., Fu S.C., Tomii K., Horton P., Imai K. (2015). Mitofates: Improved Prediction of Mitochondrial Targeting Sequences and Their Cleavage Sites. Mol. Cell. Proteom..

[19] Buchan D.W., Minneci F., Nugent T.C., Bryson K., Jones D.T. (2013). Scalable web services for the PSIPRED Protein Analysis Workbench. Nucleic Acids Res..

[20] Jamroz M., Niemyska W., Rawdon E.J., Stasiak A., Millett K.C., Sułkowski P., Sulkowska J.I. (2015). KnotProt: A database of proteins with knots and slipknots. Nucleic Acids Res..

[21] Iakoucheva L.M., Radivojac P., Brown C.J., O’Connor T.R., Sikes J.G., Obradovic Z., Dunker A.K. (2004). The importance of intrinsic disorder for protein phosphorylation. Nucleic Acids Res..

[22] Dweep H., Gretz N. (2015). miRWalk 2.0: A comprehensive atlas of microRNA-target interactions. Nat. Methods.

[23] Rabouille C. (2017). Pathways of Unconventional Protein Secretion. Trends Cell Biol..

[24] Hung M.C., Link W. (2011). Protein localization in disease and therapy. J. Cell Sci..

[25] Marunaka K., Furukawa C., Fujii N., Kimura T., Furuta T., Matsunaga T., Endo S., Hasegawa H., Anzai N., Yamazaki Y. (2017). The RING finger-and PDZ domain-containing protein PDZRN3 controls localization of the Mg2+ regulator claudin-16 in renal tube epithelial cells. J. Biol. Chem..

[26] Ferecatu I., Le Floch N., Bergeaud M., Rodríguez-Enfedaque A., Rincheval V., Oliver L., Vallette F.M., Mignotte B., Vayssière J.-L. (2009). Evidence for a mitochondrial localization of the retinoblastoma protein. BMC Cell Biol..

[27] Schaeffer C., Creatore A., Rampoldi L. (2014). Protein trafficking defects in inherited kidney diseses. Nephrol. Dial. Transplant..

[28] Scholl U., Hebeisen S., Janssen A.G., Müller-Newen G., Alekov A., Fahlke C. (2006). Barttin modulates trafficking and function of CIC-K channels. Proc. Natl. Acad. Sci. USA.

[29] Wojciechowski D., Thiemann S., Schaal C., Rahtz A., de la Roche J., Begemann B., Becher T., Fischer M. (2018). Activation of renal CIC-K chloride channels depends on an intact N terminus of their accessory subunit barttin. J. Biol. Chem..

[30] Hou J. (2012). Lecture: New light on the role of claudins in the kidney. Organogenesis.

[31] Himmerkus N., Shan Q., Goerke B., Hou J., Goodenough D.A., Bleich M. (2008). Salt and acid-base metabolism in claudin-16 knockdown mice: Impact for the pathophysiology of FHHNC patients. Am. J. Physiol. Ren. Physiol..

[32] Gong Y., Renigunta V., Himmerkus N., Zhang J., Renigunta A., Bleich M., Hou J. (2012). Claudin-14 regulates renal Ca++ transport in response to CaSR signalling via a novel microRNA pathway. EMBO J..

[33] Breiderhoff T., Himmerkus N., Drewell H., Plain A., Günzel D., Mutig K., Willnow T.E., Müller D., Bleich M. (2018). Deletion of claudin-10 rescues claudin-16-deficient mice from hypomagnesemia and hypercalciuria. Kidney Int..

[34] Chen G., Wang Y., Wang L., Xu W. (2017). Identifying prognostic biomarkers based on aberrant DNA methylation in kidney renal clear cell carcinoma. Oncotarget.

[35] Hou J., Zhao D. (2013). MicroRNA Regulation in Renal Pathophysiology. Int. J. Mol. Sci..

[36] Mladinov D., Liu Y., Mattson D.L., Liang M. (2013). MicroRNAs contribute to the maintenance of cell-type-specific physiological characteristics: MiR-192 targets Na(+)/K(+)-ATPase β1. Nucleic Acids Res..

[37] Vall-Palomar M., Arévalo J., Ariceta G., Meseguer A. (2018). Establishment of urinary exosome-like vesicles isolation protocol for FHHNC patients and evaluation of different exosomal RNA extraction methods. J. Transl. Med..

[38] Deaton A.M., Bird A. (2011). CpG islands and the regulation of transcription. Genes Dev..

[39] Beck S., Rhee C., Song J., Lee B.K., LeBlanc L., Cannon L., Kim J. (2018). Implications of CpG islands on chromosomal architectures and modes of global gene regulation. Nucleic Acids Res..

[40] Kim T.K., Shiekhattar R. (2015). Architectural and Functional Commonalities between Enhancers and Promoters. Cell.

[41] Anish R., Hossain M.B., Jacobson R.H., Takada S. (2009). Characterization of transcription from TATA-less promoters: Identification of a new core promoter element XCPE2 and analysis of factor requirements. PLoS ONE.

[42] Mehmood M.A., Sehar U., Ahmad N. (2014). Use of Bioinformatics Tools in Different Spheres of Life Sciences. J. Data Min. Genom. Proteom..

